# Neurological Impairment Linked with Cortico-Subcortical Infiltration of Diffuse Low-Grade Gliomas at Initial Diagnosis Supports Early Brain Plasticity

**DOI:** 10.3389/fneur.2015.00137

**Published:** 2015-06-10

**Authors:** Anja Smits, Maria Zetterling, Margareta Lundin, Beatrice Melin, Markus Fahlström, Anna Grabowska, Elna-Marie Larsson, Shala Ghaderi Berntsson

**Affiliations:** ^1^Department of Neuroscience, Neurology, University Hospital, Uppsala, Sweden; ^2^Department of Neurology, Danish Epilepsy Center, Dianalund, Denmark; ^3^Department of Neuroscience, Neurosurgery, University Hospital, Uppsala, Sweden; ^4^Department of Neuroradiology, University Hospital, Örebro, Sweden; ^5^Department of Radiation Sciences, Umeå University, Umeå, Sweden; ^6^Department of Radiology, Oncology and Radiotherapy, Radiology, University Hospital, Uppsala, Sweden

**Keywords:** low-grade gliomas, neurological function, professional situation, tumor volume, tumor location, brain plasticity, radiological diagnosis

## Abstract

Diffuse low-grade gliomas (DLGG) are slow-growing brain tumors that in spite of an indolent behavior at onset show a continuous expansion over time and inevitably transform into malignant gliomas. Extensive tumor resections may be performed with preservation of neurological function due to neuroplasticity that is induced by the slow tumor growth. However, DLGG prefer to migrate along subcortical pathways, and white matter plasticity is considerably more limited than gray matter plasticity. Whether signs of functional decompensating white matter that may be found as early as at disease presentation has not been systematically studied. Here, we examined 52 patients who presented with a DLGG at the time of radiological diagnosis. We found a significant correlation between neurological impairment and eloquent cortico-subcortical tumor localization, but not between neurological function and tumor volume. These results suggest that even small tumors invading white matter pathways may lack compensatory mechanisms for functional reorganization already at disease presentation.

## Introduction

Diffuse low-grade gliomas (DLGG) are slow growing primary brain tumors occurring mainly in young adults. DLGG are classified as gliomas WHO (World Health Organization) Grade II and characterized by extensive invasion but low proliferation (1). In spite of advances in diagnostic methods and surgical techniques, allowing extensive and safe tumor resections as well as the introduction of molecular tumor markers guiding therapeutic decisions, the clinical management of DLGG remains challenging (2). Sequential magnetic resonance imaging (MRI) studies of DLGG have demonstrated a linear growth in diameter of the bulky tumor mass before first-line treatment (3). In parallel with a continuous expansion over time, DLGG migrate along the white matter pathways where the invasion rate is estimated to be about five times higher than in the gray matter (4).

Around 70–90% of all patients with DLGG present with epileptic seizures and epilepsy may be the only symptom during the initial years after diagnosis (5). Some patients, however, may have focal neurological signs or cognitive impairment already at disease presentation. In general, impaired function in patients with brain tumors can be induced by compression of brain structures, either directly or indirectly by reactive edema. Additional factors affecting cognition are uncontrolled seizures, side effects of anticonvulsant treatment, and psychological distress due to brain tumor diagnosis (6).

Optimal functioning of the brain depends on intact neurological, cognitive, and affective function and is determined by the anatomic and physiological integrity of complex cerebral networks (7). The complex interactions between the tumor and the host brain are still not well understood. There is growing evidence that loss of neuronal network integrity in patients with brain tumors has a negative impact on cerebral function and decreases the threshold to develop seizures (8). The degree of functional loss appears to be related to the growth rate of the tumor, i.e., fast growing tumors cause more profound loss than slowly growing tumors. Brain plasticity most commonly refers to adaptive changes in neural pathways, synapses, and glial cells, leading to functional or morphological reorganization. Numerous surgical studies have provided support for that DLGG induce brain plasticity through functional compensation and reorganization of the cortex (9). In addition to gray matter plasticity, the subcortical pathways play a crucial role in shaping cortical reorganization (10). This concept is exemplified by the fact that large tumor resections have been performed without inducing functional loss, as long as the network connectivity is preserved (11). It is generally accepted though that the plasticity of the white matter is considerably more limited than that of the gray matter (11).

DLGG are diagnosed at various steps along the continuum of their natural course, which has been proposed to occur as a three-step process; an initial silent period, followed by a symptomatic period, and a final period of malignant progression (12, 13). In the silent phase when tumor diagnosis is still unknown, tumor-related symptoms may be present but not recognized (14). Subtle disease-related symptoms at such an early phase might nevertheless have an impact on daily life activities, including professional life. In this study, we hypothesized that patients with tumors invading white matter structures carry a risk for impairment of neurological function already at disease presentation. In other words, tumors in eloquent areas affecting subcortical pathways may cause earlier loss of function than tumors with strictly cortical location, irrespective of tumor volume. To test this hypothesis, we studied a cohort of 52 patients with DLGG at the time of radiological diagnosis and correlated their clinical parameters to tumor volume and tumor location.

## Materials and Methods

### Patients

Consecutive patients (≥18 years) presenting with a suspected DLGG at the Department of Neurosurgery, Uppsala University Hospital were enrolled during two time periods. Twenty-four patients were recruited between 2005 and 2008 from an ongoing multicenter glioma study at our hospital (15) and 28 patients were recruited between 2011 and 2013 from a previously described cohort of DLGG (16). The study methodology was similar for both patient groups and baseline clinical characteristics (mean age, gender, presenting symptoms) did not differ significantly, allowing pooling of data. The institutional review board approved the study protocols and written informed consent was obtained prior to participation. Initial inclusion criteria were morphological MRI findings suggestive of a DLGG, based on typical appearance with high signal intensity on fluid attenuated inversion recovery (FLAIR) sequence and a T1-weighted sequence showing no or only patchy and faint contrast enhancement. Inclusion criteria by central pathology review were gliomas WHO grade II (1), leaving a final study sample of 52 tumors (23 astrocytomas, 8 oligoastrocytomas, and 21 oligodendrogliomas).

### Assessment of neurological function and seizure control

Within 3 months from radiological tumor diagnosis, the neurologist performed neurological examination with special attention to language, motor and sensory function, visual fields, and cognitive function. Neurological function was rated according to the Radiation Therapy Oncology Group (RTOG) Neurological Function Status, as follows (17): 0 = no neurological symptoms; 1 = minor neurological symptoms; 2 = moderate neurological symptoms, fully active at work/home but requiring assistance; 3 = moderate neurological symptoms, less than fully active at home/work and requiring assistance; 4 = severe neurological symptoms, totally inactive requiring complete assistance at home or in institution – unable to work.

In connection with neurological examination, patients completed a questionnaire on the type of first symptoms and, in case of seizures, on seizure control during the previous 2 months. The burden of epilepsy was estimated taking into account the frequency of seizures during these 2 months and the number of antiepileptic drugs (AED), and rated as previously described with slight modification (18): 1 = no epilepsy; 2 = epilepsy but seizure-free without AED; 3 = epilepsy but seizure-free on AED; 4 = epilepsy with fewer than three seizures on AED; 5 = epilepsy with at least three seizures on monotherapy with AED, and 6 = epilepsy with at least three seizures on polytherapy with AED.

### Assessment of changes in professional life

Patients were asked to report their educational background, profession, and any changes in professional situation during the year preceding radiological diagnosis, according to a battery with standardized questions. Based on the number of years and level of higher education, patients were divided into three groups: retired (*n* = 8), professions requiring higher education (*n* = 19), and lower skilled professions (*n* = 18) (Table 1). Among higher educated patients, there were university professors, biologists, computer designers, optician, university students, clerk, teacher, businessmen, nurses, and marketing managers. Lower skilled professions comprised electricians, carpenters, construction workers, home care and hospital assistants, and housekeepers.

**Table 1:** **Patient and tumor characteristics of the cohort (*n* = 52)**.

	Number (%)	Mean ± SD	Range
**Patient characteristics**
Age (year)	52	44.8 ± 14	22–78
Gender
Male	34		
Female	18	
Seizures at presentation	38 (73.1%)		
Antiepileptic drugs	37	
Seizure-free	20	
Recurrent seizure	18	
No seizures at presentation	14 (29.9%)	
Neurological function (RTOG)	52	1.19 ± 1.15	0–3
No neurological symptoms	17 (32.7%)		
Minor neurological symptoms	21 (40.4%)	
Moderate neurological symptoms	14 (26.9%)	
Professions (*n* = 52)
High education	26 (50%)		
Low education	18 (34.6%)		
Retired	8 (15.4%)		
Work situation (*n* = 52)
Working fully	25 (48%)		
Working half time	11 (21%)		
Not working, due to illness	4 (8%)		
Retired, unrelated to illness	8 (15%)		
Students	2 (4%)		
Unemployed	2 (4%)		
Changes in professional situation	19 (36%)		
**Tumor characteristics**
Affected hemisphere
Right	20 (38.5%)		
Left	31 (38.5%)		
Bilateral	1 (1.9%)		
Tumor location	52		
Non-eloquent cortex	8		
Non-eloquent cortico-subcortical	12		
Eloquent cortico-subcortical	32		
Insula	9		
SMA^a^/Pre SMA	5/2		
Primary somatosensory area	6		
Primary motor area	4		
Language area	6		
Tumor volume (cm^3^)	50	69.3 ± 57.3	2.8–267.8
Smaller (<61 cm^3^)	24		
Larger (>61 cm^3^)	26		

*^a^Supplementary motor area*.

### Tumor volume

A neuroradiologist blinded for patient data evaluated all preoperative MRI examinations. In-house developed software was used for tumor delineation. Most DLGG appeared as an area of high signal intensity without surrounding edema on the FLAIR sequence. The entire area with high signal intensity was regarded as tumor tissue. Contours were manually drawn on FLAIR images in all slices showing visible tumor. The calculated tumor volume (cubic centimeter) was derived from the number of voxels defined as tumor multiplied with the voxel size of the image set.

### Tumor location

Tumor location was evaluated on preoperative MRI including T1-weighted images before and after gadolinium-based contrast enhancement, T2-weighted and FLAIR sequences. Locations were defined as mainly cortical or cortical with subcortical affection (=cortical/subcortical). Tumor location was further specified as eloquent or non-eloquent regions, as previously described (20). Eloquent areas consisted of insula, language areas, visual cortex, supplementary motor area (SMA), primary motor area, and sensorimotor area. Non-eloquent areas included regions in the non-dominant hemisphere, i.e., right frontopolar/prefrontal area, premotor cortex/frontal operculum, anterior/midtemporal area, and right parieto-temporo-occipital junction. Frontal and temporal pole in the left or dominant hemisphere were considered non-eloquent.

### Statistical analysis

Descriptive analysis and pie charts were used to assess patient- and tumor-related parameters. Median split was used to turn the continuous volume data into a categorical one (small versus large volume). Analysis of the correlation between tumor volume respectively tumor location with neurological function and seizure control was performed by Spearman correlation test. A *P* value <0.05 was considered statistically significant. Statistical analysis was performed using IBM SPSS Inc. (version 21).

## Results

### Neurological function and seizure control

The clinical characteristics of the study sample are shown in Table 1. The distribution of neurological function, seizure as first symptoms, and seizure control is illustrated in the Figure S1 in Supplementary Material. Most patients had no or minor neurological symptoms (RTOG scale 0–1). Epileptic seizures as initial symptom were present in 38 patients (73.1%). Of these patients, all but one was on AED; 20 patients were seizure-free during the last 2 months. Thus, 18 patients had recurrent seizures of which seven patients with less than three seizures; 11 patients had at least three seizures despite monotherapy (*n* = 6) or polytherapy (*n* = 5) with AED. Fourteen patients (26.9%) had other neurologic symptoms as presenting symptom; five presented with dizziness, four with headache, one with paresis, and two with dysphasia and memory disturbances. Two patients had incidentally discovered tumors.

### Changes in professional situation

At the time of radiological diagnosis, 25 patients were fully at work (48%), 11 half time at work (21%), 2 full-time students (4%), 2 unemployed (4%), 8 retired (15%), and 4 (8%) unable to work due to disease-related symptoms (Table 1). Regarding changes in work situation during the year preceding radiological tumor diagnosis, 19 patients (36%) reported adjusted workload (*n* = 8), reduction from full-time to half time (*n* = 7), or full-time sick leave (*n* = 4) (illustrated in Figure S1 in Supplementary Material). There was a significant correlation between change in professional situation (reduced/altered work situation, *n* = 19 versus unchanged/unrelated, *n* = 33) and neurological function (moderate symptoms, *n* = 14 versus no/minor symptoms, *n* = 38) (Spearman’s *r* = 0.40, *P* = 0.008), but not between change in professional situation and seizure control (recurrent seizures, *n* = 18 versus no seizures/seizure-free, *n* = 34).

### Tumor volume

Digital images were missing in two cases and volume was measured in 50/52 patients. Mean volume was 69.30 ± 57.34 cm^3^ (median 60.69 cm^3^, range 2.77–267.77 cm^3^). There was no significant correlation between tumor volume, neither as continuous data nor as median split values (≤61 cm^3^ versus >61 cm^3^) with neurological function or with seizure control (data not shown).

### Tumor location

**Figure 1:**
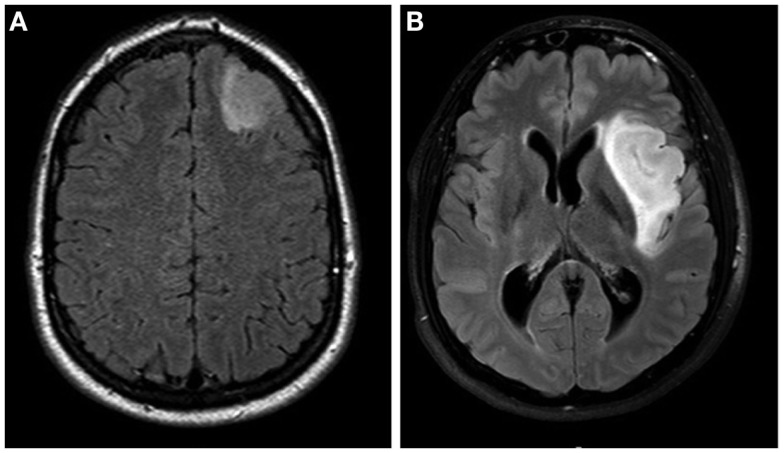
**(A)** MRI FLAIR sequence showing a left frontal astrocytoma grade II located in a non-eloquent area with mainly cortical location, in a 38-year-old female. **(B)** MRI FLAIR sequence showing an insular oligodendroglioma grade II, located affecting cortical and subcortical regions, in a 27-year-old male.

The specific tumor location is shown in Table 1. In summary, only eight tumors showed mainly cortical location, all situated in non-eloquent areas (illustrated in Figure 1A). A total of 32 tumors were situated in eloquent areas, while 20 tumors were found in non-eloquent areas. Of the 32 tumors in eloquent areas, all invaded both cortical and subcortical regions (cortico-subcortical location, illustrated in Figure 1B). Tumors in non-eloquent regions invaded frontopolar/prefrontal cortex (*n* = 11), anterior/midtemporal region (*n* = 4), right parieto-occipital area (*n* = 2), and right premotor cortex/frontal operculum (*n* = 3) (Table 1). There was a significant correlation between tumor location and neurological function when dividing tumors in eloquent cortico-subcortical (*n* = 32) versus non-eloquent cortico-subcortical/non-eloquent mainly cortical (*n* = 12 + 8 = 20) location. Thus, eloquent cortico-subcortical tumor location was correlated with impaired neurological function (Spearman’s *r* = 0.280, *P* = 0.049) in the cohort. We found no significant correlation between tumor location and seizure control (data not shown).

## Discussion

The invaded brain tissue is often regarded as a homogeneous and passive tissue modified by the tumor in a unidirectional manner. However, the slow growth and invasion of the tumor induce reciprocal processes and multiple active and passive mechanisms in the surrounding tissue (21). Consequently, patients with DLGG can undergo massive cerebral resections with significantly better neurological recovery than after acute brain injuries (22). In the present study, we evaluated neurological function and seizure control at the time of radiological diagnosis in correlation with tumor volume and tumor location. Such easily available radiological parameters may predict the clinical function of this patient group, in parallel with other neurological disorders such as stroke or traumatic brain lesions. We found a significant correlation between neurological impairment and tumor location in eloquent cortico-subcortical areas. By contrast, the grade of neurological symptoms in our cohort was not correlated with tumor volume. Our findings are consistent with the concept that reorganization of networks occurs in the silent phase of DLGG, and that tumors invading the white matter may lack such compensatory mechanisms at early phase of disease (23).

For rating of neurological function, we used the RTOG Neurological Function Scale, which is a widely used but blunt instrument. We also recorded changes in professional situation during the year preceding brain tumor diagnosis, such as adjustments in work tasks or reduced workload. From a clinical perspective, the latter parameter may present an indirect indicator for tumor-related symptoms for the patient. Interestingly, a high proportion of patients (36%) in our cohort encountered a change in professional situation during the year prior to tumor diagnosis. This percentage is higher than what is to be expected in the normal working population in our country (24). These findings suggest that reduced or lost professional capacity due to disease-related symptoms may precede radiological tumor diagnosis and underscore the importance of evaluating this aspect at first visit to the clinic. Our results also argue for early diagnostic intervention of individuals with slow onset difficulties to cope with work or studies without any clear explanatory factors. There was no difference in professional situation between patients with high- and low-skilled professions, which may reflect the relatively small numbers in the different subgroups. In this context, it is important to bear in mind that our study is based on limited clinical data and that the data presented here are preliminary findings that need confirmation in larger studies. This is also exemplified by the cutoff value for tumor volume (61 cm^3^) calculated by using median split, which is relatively small compared to what is known from the European Organization for Research and Treatment of Cancer (EORTC) trials on DLGG (25).

One study so far has demonstrated that impairment of specific neurological function is an indicator for the professional reintegration of patients with DLGG (26). In contrast to patients with normal language function after tumor resection in language areas, those with impaired lexicon access speed were not able to return to work (26). Since neuropsychological test results were available only for a small subset of patients, these data could not be included in our analysis. The lack of neuropsychological test results is an obvious limitation of the present study and, together with the small sample size, a strong argument for the need of future trials including specific cognitive test batteries (19).

Epileptic seizures were the first tumor manifestation in the majority of patients in this study and all but one patient with seizures were on AED. In agreement with a recent multicenter study including 1509 patients, we did not find a correlation between tumor volume and seizure control (5). Only 11 patients in our cohort had more than three seizures during the previous 2 months, of whom five used at least two AED. The relatively good seizure control may reflect the early phase of the disease but also better compliance for new generation AED compared to older AED. Most patients in our cohort were on new AED such as levetiracetam and lamotrigine that are now routinely used in the brain tumor population because of their favorable adverse effect profile (27).

In conclusion, the data presented here show that patients with DLGG in eloquent areas invading subcortical pathways may be at risk for neurological impairment already at the time of radiological diagnosis. Our findings warrant well-designed future trials evaluating neurological and cognitive function in this early phase of the disease.

## Conflict of Interest Statement

The authors declare that the research was conducted in the absence of any commercial or financial relationships that could be construed as a potential conflict of interest.

## Supplementary Material

The Supplementary Material for this article can be found online at http://journal.frontiersin.org/article/10.3389/fneur.2015.00137/abstract
