# Supplementary figures and images for: Neurological Impairment Linked with Cortico-Subcortical Infiltration of Diffuse Low-Grade Gliomas at Initial Diagnosis Supports Early Brain Plasticity
Source: Front Neurol. 2015 Jun 10;6:137. doi: 10.3389/fneur.2015.00137 (PMC4462100; doi:10.3389/fneur.2015.00137)

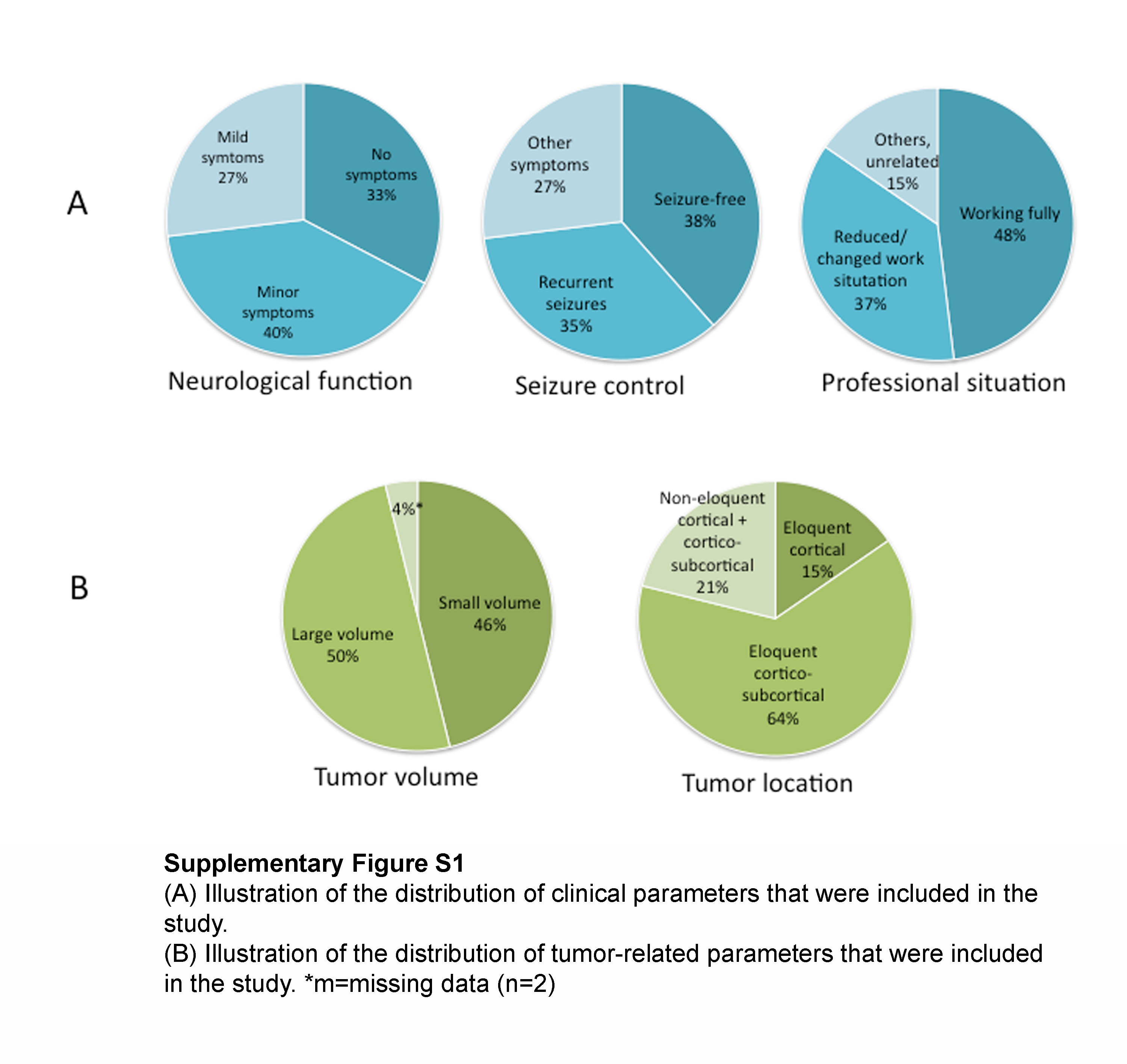

Supplement: Supplementary file 1 [file Image_1.TIF]
